# Interleukin 10 and interleukin 10 receptor in paediatric inflammatory bowel disease: from bench to bedside lesson

**DOI:** 10.1186/s12950-021-00279-3

**Published:** 2021-03-10

**Authors:** Paulina Krawiec, Agnieszka Pawłowska-Kamieniak, Elżbieta Pac-Kożuchowska

**Affiliations:** Department of Paediatrics and Gastroenterology, Medical University of Lublin, Racławickie 1, 20-059 Lublin, Poland

**Keywords:** Crohn’s disease, Genetics, Immunity, Ulcerative colitis

## Abstract

**Background:**

The differences between adults and children in inflammatory bowel disease (IBD) phenotype, severity, complications, co-morbidities, and response to the therapy resulted in the extraction of paediatric IBD. It has been revealed that the substantial role in the development of IBD in children under 6 years of age plays a single genetic mutation (monogenic IBD). On the other hand, in older children and adolescents IBD is usually associated with number of interactions between susceptibility loci (polygenic IBD).

**Main body:**

Until now there have been described about 60 monogenic defects which affect the variety of immune mechanisms in IBD pathogenesis including epithelial barrier, function of neutrophil granulocytes and phagocytes, T- and B-cell selection and activation, immune inhibitory mechanisms, or apoptosis. Il-10 is an anti-inflammatory cytokine which modulates innate and adaptive immunity affecting expression of pro-inflammatory molecules and function of the variety of immune cells. Patients with identified defects in Il-10 pathway manifest with life-threating colitis with perianal lesions which occurs within first months of life. Allogenic hematopoietic stem cell transplantation is curative therapy in children with Il-10 signalling defects.

**Conclusion:**

Clinical awareness of Il-10 signalling defects enables early recognition and prompt management of the disease.

## Background: overview of paediatric inflammatory bowel disease

Inflammatory bowel disease (IBD) is a group of disorders characterized by a chronic relapsing inflammation of gastrointestinal tract. Pathogenesis of IBD involves a complex interplay between genetic susceptibility, environmental factors, dysbiosis and immune dysregulation [1]. However, the exact mechanisms of IBD development remain unclear.

The differences in IBD phenotype, severity, complications, co-morbidities and response to the therapy between adults and children resulted in the extraction of paediatric IBD [2]. Paediatric onset of IBD refers to patients with the disease onset prior to 17 years of age. According to patients’ age at IBD onset paediatric IBD may be subdivided into:

- early onset IBD (EOIBD) recognized in children younger than 10 years of age,
- very early onset IBD (VEOIBD) recognized in children younger than 6 years of age,
- infantile (toddler) onset IBD recognized in children younger than 2 years of age and
- neonatal IBD recognized in children during the first 28 days of age [2].

It has been observed that a single genetic mutation plays the substantial role in the development of very early onset of IBD (monogenic IBD) [2, 3]. On the other hand, in older children and adolescents the overall risk of IBD is associated with number of interactions between susceptibility loci [2, 3]. Kaser et al. suggested that polygenic IBD development is significantly affected by environmental factors [3].

That fact may explain diversity in IBD phenotype between children with VEOIBD from those with older-onset IBD and may lead to identification of novel target therapeutic options and tailored-made approach to paediatric IBD patients [4].

Until now there have been described about 60 monogenic defects [5] which affect the variety of immune mechanisms in IBD pathogenesis including epithelial barrier, function of neutrophil granulocytes and phagocytes, T- and B-cell selection and activation, immune inhibitory mechanisms or apoptosis [2, 5]. The aim of our study is to present clinical aspects of defects in Il-10 signalling in children with early onset of IBD.

## Interleukin-10 signalling pathway

Interleukin 10 (Il-10) is a member of Il-10 cytokine family, comprising Il-19, Il-20, Il-22, Il-24, Il-26, Il-28 and Il-29, which acts through a type II cytokine receptors [6, 7].

Il-10 is encoded by the *Il-10* gene located on the long arm of chromosome 1 (region 1q32) [8]. Il-10 is a 36 kDa homodimer of two 160 amino acid polypeptide chains [9]. Each subunit consists of six helices which are connected by two loops and three turns [7, 9]. Il-10 is expressed by the wide range of innate and adaptive immune cells including T and B lymphocytes, monocytes, macrophages, natural killer cells (NK), neutrophils, mast cells, dendritic cells and epithelial cells [6].

Il-10 binds to the tetrameric receptor (Il-10R) which is formed of two α-chains of Il-10 receptor 1 (Il-10R1) and two β-chains of Il-10 receptor 2 (Il-10R2) [7, 9–11]. Each of these chains consists of extracellular, transmembrane and intracellular domain [9]. Il-10 receptor 1 is specific for Il-10 binding, while Il-10 receptor 2 may be a signal transducing subunit of other representatives of Il-10 cytokine family [11, 12].

The binding of Il-10 to its receptor activates Janus kinase 1 (JAK1) and tyrosine kinase 2 (Tyk2). Both kinases phosphorylate Il-10R1 which subsequently phosphorylates and recruits signal transducer and activator of transcription 3 (STAT3). Phosphorylation of STAT3 result in its dimerization, translocation to the nucleus and promotion of target genes’ transcription. Eventually a down-stream signalling cascade leads to expression of anti-inflammatory effectors [8, 10–13].

Il-10 is a crucial anti-inflammatory cytokine which modulates innate and adaptive immunity affecting expression of proinflammatory molecules and function of the variety of immune cells. Il-10 inhibits the maturation and antigen-presenting cell function of dendritic cells through suppression of Iκβ kinase (IKK), serine-threonine kinase Akt and nuclear factor kappa-light-chain-enhancer of activated B cells (NFκβ) [14]. Il-10 produced by dendritic cells causes ubiquitination and degradation of myeloid differentiation factor (MyD)88-dependent signalling molecules including Il-1 receptor-associated kinase IRAK4, IRAK1 and TNF-receptor associated factor (TRAF)6, to inhibit TLR-mediated MyD88-dependent pathway [15]. That process contributes in the negative regulation of the expression of inflammatory cytokines including Il-6, Il-1β and TNF-α [16]. Il-10 suppresses Th1/Th17 cell-mediated adaptive immune response [16, 17]. In response to Il-10 the expression of major histocompatibility complex II (MHC class II) is reduced on monocytes, macrophages and dendritic cells and increased on B lymphocytes [8, 16]. Moreover, Il-10 enhances secretion of immunoglobulins and immunoglobulin class-switching in B cells [16].

Although Il-10 down-regulates expression of inflammatory mediators and inhibits antigen presentation, it stimulates proliferation and up-regulates activation of NK-cells, CD8+ T-cells, B lymphocytes and mast cells [8, 16, 18]. Table 1 presents summary of main functions of Il-10 in host immune response [8, 16, 18, 19].

**Table 1:** Summary of Il-10 main functions in immune response

Target cells	Effect of Il-10
B cells	Enhancement of proliferation and activation [18] Stimulation B cell differentiation into plasma cells [16] Induction of class switch to IgA, IgE, IgG1 and IgG3 and antibody production [16]
CD4^+^ T cells	Suppression of proliferation and activation [19]
CD8^+^ T cells	Stimulation of proliferation and cytotoxic activity [19]
Dendritic cells	Interference in the maturation process of DCs [16] Downregulation of MHC class II and co-stimulatory molecules CD80/CD86 expression [8, 16] Inhibition of Il-12 production [16]
NK cells	Stimulation of proliferation and cytotoxic activity [19]
Macrophages	Downregulation MHC class II and co-stimulatory molecules CD80/CD86 expression [16] Suppression of NO generation [16] and production of proinflammatory cytokines Il-1, Il-6, TNFα, and Il-12 [19] Inhibition of autophagy induction [16]

## Interleukin-10 knockout mouse model of inflammatory bowel disease

In the past decades, it has been shown that Il-10 deficient mice with lacking functional Il-10 (*Il-10*^*−/−*^) or Il-10 R2 subunit (*Il-10 Rb*^*−/−*^) developed spontaneous enterocolitis [20, 21] which resulted from unbalanced Th1/Th17 response, excessive release of proinflammatory cytokines (mainly Il-12, Il-13, Il-17, Il-23, IFNγ) and failure in homeostatic relationship between microbiota and the host [16, 17].

Interleukin-10-deficient mice colitis is a multi-hit model of colitis which involves genetic factor, immune disturbances and alterations of gut microbiota [17]. It has been shown that the presence of resident enteric bacteria is essential for the development of spontaneous colitis in Il-10-deficient mice, while germfree Il-10-deficient mice had no evidence of colitis or immune system activation [17, 22]. Specific pathogens including *Enterococcus faecalis*, *Escherichia coli*, *Helicobacter hepaticus* or *Murine Norovirus* appear to act as colitogenic triggers which promote inflammation in Il-10-deficient mice [17, 23–26]. Nevertheless, *Lactobacillus salivarius* 433,118 and *Bifidobacterium infantis* 35,624 were found to attenuate colitis in Il-10-deficient mice [27]. There are also known some strain-specific genetic factors which may determine mucosal immune response [17]. For example, it has been reported that a major colitogenic loci *Cdcs1* may modify the response to inflammation induced by *Helicobacter hepaticus* in the murine model [25]. On the other hand, dysbiosis may also alter strain-specific colitis susceptibility and host immune response [17, 25].

In conventional environment chronic enterocolitis in Il-10 deficient mice occurs by 2–3 months of age, soon after weaning and features transmural involvement and mucosal discontinuity [17, 20, 28]. Inflammatory cells including lymphocytes, plasmocytes, macrophages, eosinophils, and neutrophils infiltrate into the lamina propria and submucosa. Other pathological lesions of enterocolitis in Il-10 deficient mice are as follows epithelial hyperplasia, mucin depletion, crypt abscesses, ulcers, and thickening of bowel wall [17, 20, 28].

## Interleukin-10 pathway defects in humans

Defects of Il-10 and Il-10 receptors are monogenic autosomal recessive diseases with 100% penetrance [2, 29]. Since first description of mutations in genes *Il-10RA* and *Il-10RB* in patients with early-onset colitis in 2009, about 70 cases of Il-10/Il-l0R deficient patients with IBD phenotype have been identified [11–13]. Although the frequency of Il-10 and Il-10 receptor defects is not established, several studies reported data on their prevalence in cohorts of children with IBD [30, 31]. Among 66 children with early-onset of IBD there were identified 16 (24.2%) with mutations in Il-10-related genes including 8 (12.1%) with mutation in *Il-10RB* gene, 5 (7.6%) with mutation in *Il-10RA* gene and 3 (4.5%) with mutation in *Il-10* gene [30]. Genetic screening in a group of 62 IBD children with disease onset before the age of 2 years revealed 5 (8%) cases of *Il-10* or *Il-10R* genes’ mutations i.e., 2 (3.2%) with *Il-10* gene mutation, 2 (3.2%) with *Il-10RB* gene mutation and 1 (1.6%) with *Il-10RA* gene mutation [31].

Defects in Il-10 signalling pathway manifest as a Crohn’s disease-like conditions [2, 12]. Although no clear genotype-phenotype correlations have been established, there are some differences in clinical picture of Il-10 and Il-10R defects. Patients with identified mutations in *Il-10* gene in the first 3 months of life presented with colitis with perianal lesions which was resistant to complex therapy approach (steroids, immunosuppression, anti-TNF-alfa agents). The disease is manifested as a severe bloody diarrhoea which may lead to the weight loss and growth impairment [12, 32]. Patients suffer also from the presence of perianal fistulas or abscesses [12, 32]. One patient with Il-10 deficiency experienced also oral ulcers and moderate hearing loss [12]. In general children with Il-10 deficiency did not exhibit any extraintestinal manifestations [2, 12]. Among patients with mutations in *Il-10* gene there were no significant alterations in the level of immunoglobulins, apart from increased level of IgA and IgM in one case [12, 30].

Children with mutations in *Il-10RA* and *Il-10RB* genes manifested with severe, intractable granulomatous enterocolitis with perianal lesions and penetrating behaviour within first months of life [12, 30]. In contrast to Il-10 deficiency, Il-10 receptor deficiencies are associated with some extraintestinal manifestations like folliculitis, eczema, arthritis, or autoimmune hepatitis [2, 12, 30]. Possible causes of the fact that folliculitis may indicate Il-10RB deficiency include the fact that beta chain of Il-10 receptor is expressed on keratinocytes and secondly it may be a signal transducing subunit of the receptors for other interleukins [12, 32].

Recurrent infections were frequently noted in that group of patients [33]. There have been also described cases of children with Il-10RB defect who revealed B-cell lymphoma [12, 30]. It has been suggested that since IL-10RB is a part of λ-interferon involved in antiviral defence its deficiency and immunosuppressive therapy may increase risk of viral infections and EBV-induced lymphomas [12, 32].

Several patients with mutations in *Il-10RA* or *Il-10RB* genes had increased level of immunoglobulins IgA, IgG or IgM [12].

Similarly to IBD associated with mutations in *Il-10* gene, Il-10 receptor deficiencies are also refractory to conventional immunosuppressive therapy [12, 30]. To date the only effective documented curative therapy in children with Il-10 signalling defects is allogenic hematopoietic stem cell transplantation [2, 12, 30, 33].

It must be also noted that there have been reported several polymorphisms in Il-10 gene which are associated with increased risk of IBD in adults [8, 34]. However, this issue is beyond the scope of this review.

## Interleukin 10 - is it a therapeutic option in inflammatory bowel disease?

Since Il-10 appears to play a key role in regulation of inflammatory mediators and antigen presentation, it has been suggested as a potent anti-inflammatory therapy in IBD. Although some studies on recombinant human Il-10 reported clinical and endoscopic improvement as a result of Il-10 supplementation [35, 36], the systematic review by Buruiana et al. showed that Il-10 is not effective in the induction of remission in Crohn’s disease [37].

Several potential reasons of lack of Il-10 efficacy have been proposed including low stability and poor bioavailability of the drug [38]. Another explanation of this phenomenon may be that immunosuppressive action of Il-10 is revealed only under certain conditions or counterbalanced by its proinflammatory properties [8, 38]. It has been also suggested that Il-10 may not have ability to reverse IBD, but it may prevent IBD development [8, 38]. Last but not least, Il-10 may be insufficient to counter impaired immune response in IBD [8]. These aspects may be addressed in future studies on Il-10 pathway as a therapeutic target in IBD.

## Conclusion

Monogenic genes’ defects appear to be causal factors underlying pathogenesis of paediatric IBD with early and very early onset. There are described about 60 monogenic defects which affect the variety of immune mechanisms in IBD pathogenesis including defects in Il-10 signalling [5]. Il-10 is a crucial anti-inflammatory cytokine which modulates innate and adaptive immunity affecting expression of proinflammatory molecules and function of the variety of immune cells. Patients with identified defects in Il-10 pathway manifest with severe colitis with perianal lesions which occurs within first months of life and is resistant to standard therapy. Clinical awareness of Il-10 signalling defects enables early recognition and prompt treatment of the disease.

## Data Availability

Not applicable.

## Abbreviations

EOIBD: Early onset inflammatory bowel disease
IKK: Iκβ kinase
IBD: Inflammatory bowel disease
Il-10: Interleukin 10
Il-10R: Interleukin 10 Receptor
IRAK: I1–1 receptor-associated kinase
JAK1: Janus kinase 1
MHC class II: Major histocompatibility complex II
NK: Natural killer cells
NFκβ: Nuclear factor kappa-light-chain-enhancer of activated B cells
STAT3: Signal transducer and activator of transcription 3
TRAF: TNF-receptor associated factor
Tyk2: Tyrosine kinase 2
VEOIBD: Very early onset of inflammatory bowel disease
